# Impact of compounded financial collapse and armed conflict on access to radiation therapy in Lebanon: lessons for building resilient cancer care systems in fragile settings

**DOI:** 10.3389/fonc.2026.1836449

**Published:** 2026-08-10

**Authors:** Caroline Samaha Jabbour, Layth Mula-Hussain, Yavuz Anacak, Fadi El-Karak, Georges Fares, Toufic Eid, Hady Ghanem, Maria Pia Ibrahim, Christelle Saad, Maroun Khater

**Affiliations:** 1 Department of Radiation Oncology, Mount Lebanon Hospital, Hazmieh, Lebanon; 2 Department of Radiation Oncology, Lebanese American University Medical Center – Rizk Hospital (LAUMC-RH), Beirut, Lebanon; 3 Department of Radiation Oncology, BC Cancer Centre for the North, Prince George, BC, Canada; 4 Faculty of Medicine, University of British Columbia, Vancouver, BC, Canada; 5 Department of Radiation Oncology, Faculty of Medicine, Ege University, İzmir, Türkiye; 6 Department of Hemato-Oncology, Hôtel-Dieu de France University Hospital; School of Medicine, Saint-Joseph University, Beirut, Lebanon; 7 Laboratory of Mathematics and Applications, Faculty of Sciences, Saint-Joseph University, Beirut, Lebanon; 8 Department of Radiation Oncology, American University of Beirut Medical Center (AUBMC), Beirut, Lebanon; 9 Division of Hematology-Oncology, Lebanese American University Medical Center – Rizk Hospital (LAUMC-RH), Beirut, Lebanon; 10 Gilbert and Rose-Marie Chaghoury School of Medicine, Lebanese American University, Byblos, Lebanon; 11 Faculty of Business Administration, Saint Joseph University of Beirut, Beirut, Lebanon

**Keywords:** access to care, armed conflict, economic crisis, fragile settings, health system resilience, hypofractionation, Lebanon, radiation therapy

## Abstract

**Background:**

Lebanon’s radiation therapy (RT) system has been exposed to successive, overlapping crises since 2019, including financial collapse, the Beirut Port explosion, and armed conflict in 2024. This study analyzes the impact of compounded crises on RT access and practice patterns using longitudinal institutional data from Mount Lebanon Hospital (MLH) as a sentinel cohort.

**Methods:**

A retrospective observational study was conducted at MLH (2018–2025). Annual treated patient volumes, treatment technique distributions (3D-CRT, IMRT, SBRT, SRS), and hypofractionation adoption patterns were analyzed. Crisis phases were defined as: pre-crisis baseline (2017–2018), acute economic collapse (2019–2020), subacute adaptive phase (2021–2022), and chronic/war-related phase (2023–2025). National RT workforce and infrastructure data were assessed against ESTRO-HERO and IAEA benchmarks.

**Results:**

RT patient volume exhibited non-linear evolution: initial increase from 1,186 (2018) to 1,498 (2022), a modest decline to 1,328 (2023), with recovery to 1,399 (2024) and 1,479 (2025) coinciding with war-related patient centralization. A sustained shift occurred from 3D-CRT (>80% in 2018) to IMRT, SBRT, and SRS (>50% by 2025). Hypofractionated breast radiotherapy increased from 34.1% (2023) to 73.0% (2025), with a marked inflection point in 2024. Lebanon’s RT workforce falls below ESTRO benchmarks for radiation therapists (3.9 vs. 5–7 per LINAC) and medical physicists (0.8 vs. 1.2–1.7 per LINAC).

**Conclusion:**

RT systems in fragile settings do not fail linearly but fluctuate under sustained strain, and apparent volume stability at a single resilient center can mask systemic national contraction. Crisis conditions accelerated hypofractionation adoption and technique modernization. We further identify the need for a structured Radiation Therapy System Resilience Score (RT-SRS) and define its candidate domains as a conceptual, hypothesis-generating framework to guide proactive vulnerability detection in fragile settings; its operationalization and validation are designated as future work.

## 1 Introduction

Radiation therapy (RT) is a cornerstone of modern oncology, required in the management of approximately half of all cancer patients and contributing to approximately 40% of curative outcomes worldwide (1, 2). Unlike other oncologic interventions, RT is uniquely dependent on uninterrupted daily delivery, highly specialized multidisciplinary teams, complex capital equipment requiring continuous maintenance, and stable financial and regulatory environments (3, 4). These characteristics render RT particularly vulnerable to systemic shocks, especially in fragile health systems and conflict-affected settings.

Global oncology literature has increasingly addressed cancer care in low- and middle-income countries (LMICs) and humanitarian contexts (5, 6). However, most analyses focus on isolated crises or short-term disruptions. Far less attention has been paid to the cumulative and synergistic effects of overlapping crises, particularly when chronic economic erosion precedes and amplifies the impact of acute shocks such as armed conflict.

Lebanon offers a unique and instructive model for studying these dynamics. Since 2019, the country has experienced a cascade of compounding crises: a catastrophic financial collapse, the COVID-19 pandemic, the Beirut Port explosion, and armed conflict escalating in 2024. These events unfolded within a health system that was structurally fragmented, heavily privatized, and weakly governed, yet technically advanced in several areas of tertiary care, including radiation oncology (7, 8).

This study analyzes the impact of compounded crises on access to and quality of radiation therapy in Lebanon, using longitudinal institutional data from Mount Lebanon Hospital as a sentinel cohort. Beyond documenting disruption, it aims to reframe resilience, not as heroic endurance by individual practitioners, but as a property of systems deliberately designed to withstand instability. We further propose a structured Radiation Therapy System Resilience Score (RT-SRS) as a proactive planning tool for fragile settings.

## 2 Background

### 2.1 Lebanon as a paradoxical health system

Lebanon is a small Eastern Mediterranean country with a population of approximately 5.5–6 million and one of the highest refugee-to-population ratios globally (9, 54, 55). Its confessional power-sharing political system results in chronic institutional fragmentation, weak central governance, and recurrent political instability (10).

Prior to 2019, Lebanon was classified as an upper-middle-income economy with advanced tertiary healthcare. In 2019, it ranked 23rd globally on the Bloomberg Healthcare Efficiency Index (score 53.0; life expectancy 79.4 years) (11). The Global Health Security Index 2021 indicated moderate emergency preparedness (12), while the Numbeo Health Care Index 2025 placed Lebanon 51st globally (score 63.7) (13). Simultaneously, Lebanon exhibited structural fragility: limited public healthcare provision, heavy private-sector dependence, and pervasive institutional corruption (14, 15). This coexistence of advanced clinical capacity and weak governance created a paradoxical baseline, capable of high-quality care, yet poorly equipped to absorb sustained shocks (7).

Cancer represents a growing public health burden. According to the Global Burden of Disease Study 2021, Lebanon experienced among the fastest increases in cancer incidence globally, with new cases rising approximately 162% from 1990 to 2023 (16, 48). During the crisis period, the National Cancer Registry had not been publicly updated beyond 2015 (17, 57), severely limiting real-time surveillance and planning capacity; site-specific incidence data for 2016–2024 were released only retrospectively in late 2025 (18).

### 2.2 Radiation therapy landscape prior to 2019

The geographic distribution of Lebanon’s RT centers is illustrated in **Supplementary Figure 1**. Before the financial crisis, Lebanon hosted approximately 12 RT centers, predominantly private and geographically concentrated in Beirut and Mount Lebanon (19). Based on ESTRO/QUARTS benchmarks (20, 21, 58), Lebanon’s equipment density of approximately 4 LINACs per million inhabitants appeared numerically adequate. However, this masked critical structural fragility: a significant proportion of installed equipment exceeded 10 years of age, and no national LINAC replacement schedule existed.

**Figure 1:**
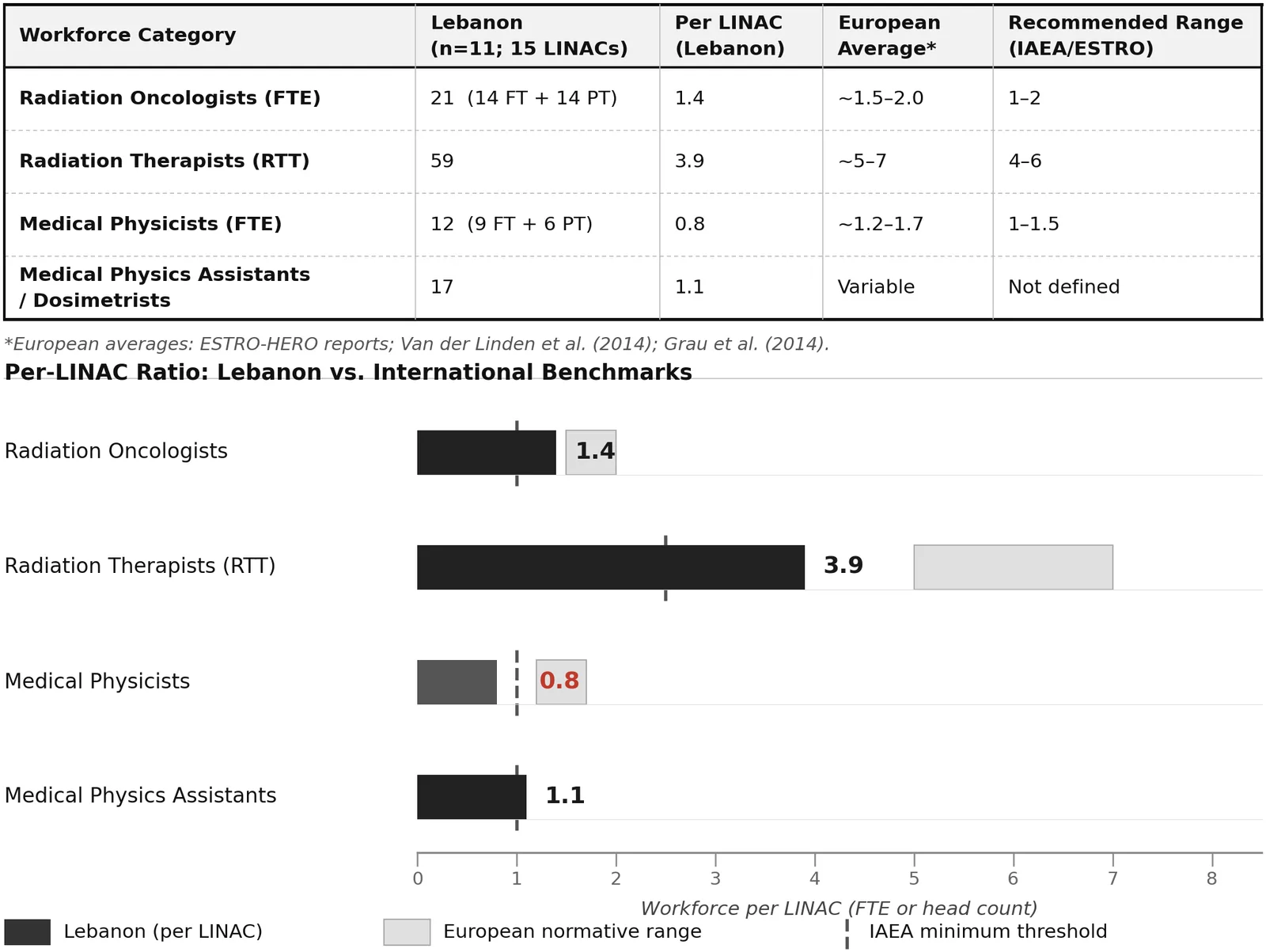
Radiation therapy workforce per LINAC: Lebanon compared to ESTRO-HERO European benchmarks. Comparison of radiation oncologist (RO), radiation therapist (RTT), and medical physicist (MP) staffing ratios per linear accelerator (LINAC) between Lebanon and ESTRO-HERO recommended ranges. Lebanon falls below benchmarks for RTTs and medical physicists, representing the primary structural workforce deficit. ESTRO, European Society for Radiotherapy and Oncology; HERO, Health Economics in Radiation Oncology.

Lebanon’s RT workforce fell below international standards. Radiation oncologist staffing (approximately 1.4 FTE per LINAC) approached the lower range of European averages (22), while radiation therapist coverage (3.9 vs. 5–7 per LINAC) and medical physicist coverage (0.8 vs. 1.2–1.7 per LINAC) fell clearly below ESTRO-HERO benchmarks (21, 22) (**Figure 1**). Critically, 50% of radiation oncologists and 40% of medical physicists were employed part-time, and no nationally accredited training or certification pathways existed.

Despite modern equipment, RT access was estimated below 40% nationally, with geographic centralization, high out-of-pocket costs for advanced techniques, and absence of structured quality assurance outside university-affiliated centers (23, 50, 59).

## 3 Definition of the crises

### 3.1 The financial and economic crisis (2019–present)

Beginning in late 2019, Lebanon entered one of the most severe financial crises globally since the mid-19th century. Currency devaluation exceeding 90%, banking sector collapse, and hyperinflation led the World Bank to reclassify Lebanon from upper-middle- to lower-middle-income economy (24, 25). Healthcare worker salaries lost most of their real value (26), insurance coverage collapsed, imports of medical equipment were disrupted, and hospital reimbursements became chronically delayed (8, 27).

The impact on oncology care was measurable: 31.87% of surveyed patients experienced treatment interruptions primarily due to drug shortages, and 69.60% were compelled to self-pay despite holding third-party insurance (28). A multicentric study found that 49.6% encountered difficulties obtaining chemotherapy and 31.6% experienced treatment deferrals (29). Among patients who altered their regimens, 89.66% cited financial difficulties as the primary driver, and one-third exhibited symptoms of anxiety or depression (28).

### 3.2 The COVID-19 pandemic and Beirut port explosion (2020–2021)

Although the COVID-19 pandemic and the August 2020 Beirut Port explosion further strained healthcare delivery through temporary service disruption, workforce fatigue, and resource diversion (27, 30), their impact on radiation therapy access was relatively limited compared with the prolonged economic collapse and the 2024 armed conflict, and therefore falls outside the primary scope of this analysis.

### 3.3 The armed conflict (2024)

In September 2024, Lebanon experienced a destructive armed conflict, with intense bombardment concentrated in southern regions, the Bekaa Valley, and southern Beirut suburbs. Although no RT department sustained direct structural damage, two centers in South Lebanon and the Bekaa were forced to suspend operations. This conflict escalated upon an already severely debilitated system, amplifying pre-existing vulnerabilities (31, 32, 51).

## 4 Materials and methods

### 4.1 Study design and setting

This retrospective observational study was conducted at Mount Lebanon Hospital (MLH), a tertiary private referral center in Hazmieh (Greater Beirut), Lebanon. MLH hosts one of the largest radiation oncology departments in the country, equipped with three LINACs and delivering the full spectrum of contemporary techniques (3D-CRT, IMRT, VMAT, SBRT, SRS). The department treats more than 1,200 patients annually and contracts with all major Lebanese payer types. Referrals originate from all 8 Lebanese governorates, providing a geographically heterogeneous cohort, although the centre is not statistically representative of national RT activity.

### 4.2 Study period and data collection

Departmental records were retrospectively reviewed for 2018–2025, encompassing pre-crisis baseline, acute economic collapse, subacute adaptive, and war-escalation phases. Collected variables included: (1) annual number of patients treated; (2) treatment technique distribution (3D-CRT, IMRT, SBRT, SRS), expressed as the proportion of treated courses per technique; the treated course, rather than the individual patient, was used as the unit of analysis, since a patient may receive more than one course of radiotherapy; (3) detailed yearly fractionation schedules for breast cancer patients (conventional, moderate hypofractionation, ultra-hypofractionation), selected as an illustrative example given that breast cancer represents one of the most prevalent malignancies in Lebanon and the most extensively documented site for hypofractionation adoption. National RT workforce data were assessed against ESTRO-HERO benchmarks.

### 4.3 Data analysis

Annual proportions were calculated and plotted to assess temporal trends. Inflection points corresponding to major crisis milestones were identified. Monthly patient volumes were additionally examined to characterize within-year fluctuations. No inferential time-series or change-point modeling was performed; given the overlapping, compounded nature of the crises and the annual granularity of several series, the identified inflection points are presented as descriptive orientation rather than as statistically demonstrated change points. To assess whether the national cancer case-mix shifted over the study period, a secondary analysis was performed on site-specific incidence data published on the Lebanese Ministry of Public Health website (National Cancer Registry, Epidemiological Surveillance Program, 2018–2024). Workforce data were compared against ESTRO-HERO benchmarks for radiation therapists and medical physicists per LINAC (21, 22). This study was conducted as a retrospective institutional audit of anonymized aggregate data; individual patient consent was not required.

## 5 Results

### 5.1 Evolution of radiation therapy activity (2018–2025)

**Figure 2:**
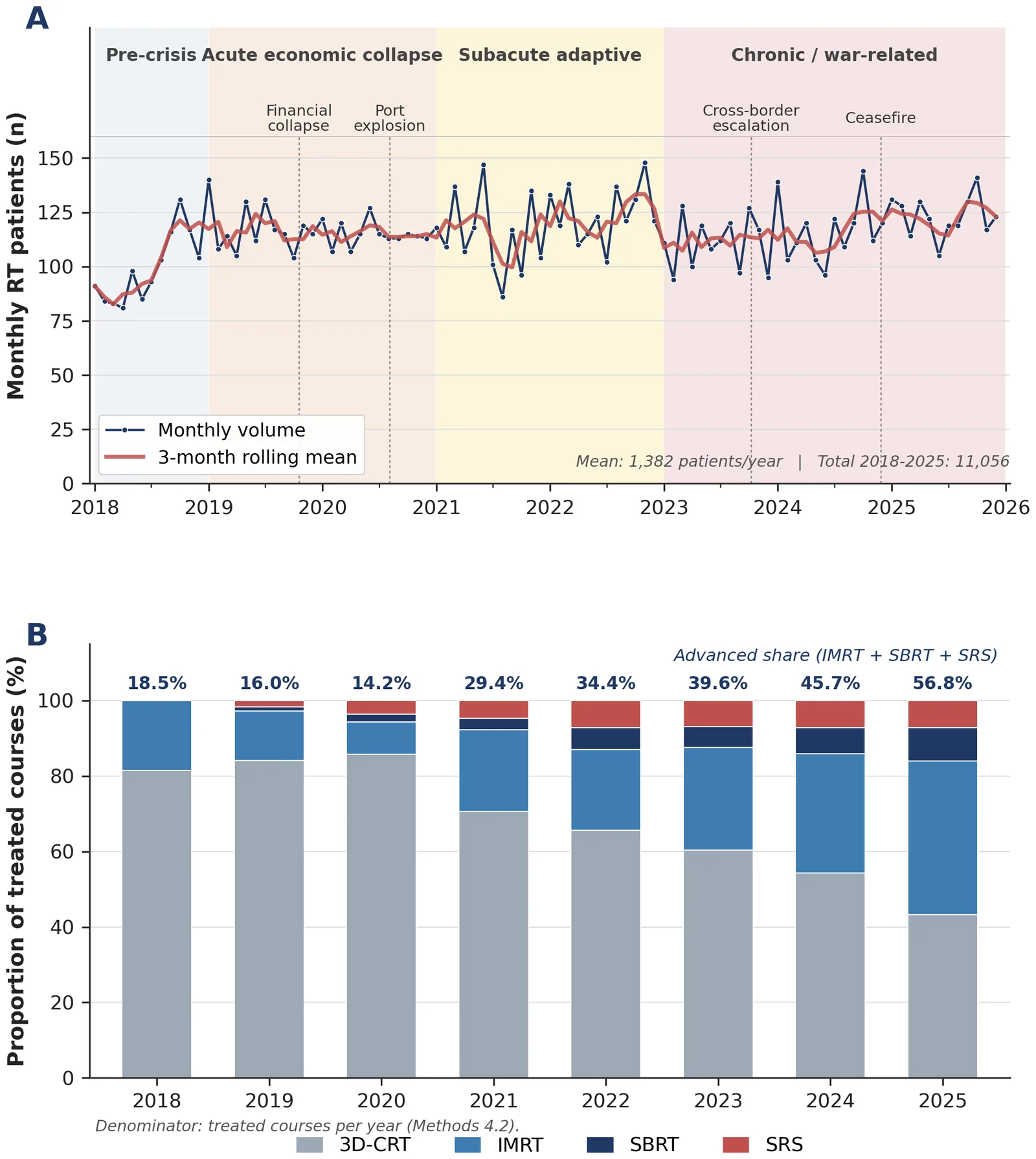
Evolution of RT patient volume and technique distribution at Mount Lebanon Hospital (2018–2025). **(A)** Monthly RT patient volume (n = 96 months; total 11,056 patients), with the four crisis phases indicated by background shading and major events marked by vertical dashed lines. The three-month rolling mean (red) clarifies the underlying trajectory: a pre-crisis rise through 2018; a plateau across the acute economic collapse and subacute adaptive phases; a modest dip in 2023; and recovery during 2024–2025, the latter coinciding with armed conflict and patient redistribution onto MLH. **(B)** Annual distribution of treated courses by technique (3D-CRT, IMRT, SBRT, SRS); the combined advanced-technique share (IMRT + SBRT + SRS) is annotated above each bar. After a brief contraction during the acute economic collapse phase (2019–2020), advanced techniques expanded markedly from 2021 onward, reaching 56.8% by 2025. No inferential time-series modeling was performed; phase boundaries and event markers are descriptive. IMRT, intensity-modulated radiation therapy; SBRT, stereotactic body radiotherapy; SRS, stereotactic radiosurgery.

MLH data reveal a non-linear evolution of RT activity closely mirroring successive crisis phases (**Figure 2**). In 2018, MLH treated 1,186 patients, reflecting a stable pre-crisis baseline. RT utilization increased markedly from 2019 (1,410 patients) through 2022 (1,498 patients), despite macroeconomic collapse, reflecting delayed pricing rescaling (23), shortages of systemic therapies driving compensatory RT expansion (28, 29), and increased reliance on RT for curative and palliative indications.

In 2023, activity declined modestly to 1,328 patients, amid cumulative workforce attrition, maintenance delays, and patient financial exhaustion, before recovering to 1,399 patients in 2024 and 1,479 patients in 2025. The 2024–2025 recovery coincided with armed conflict and the closure of RT centres in the south and the Bekaa, and is best explained by emergency centralization and patient redistribution onto MLH rather than by systemic recovery or preserved national capacity. Notably, the relatively contained dip at MLH should not be read as national stability: as a single private tertiary center that absorbed patients displaced from contracting or closing services, MLH is more likely to have masked than to have mirrored the national trajectory.

### 5.2 Transformation of treatment technique distribution

In 2018, more than 80% of treatments were delivered using 3D-CRT, with advanced techniques (IMRT, SBRT, and SRS combined) accounting for 18.5% of treated courses. During the acute economic collapse phase (2019–2020), the advanced-technique share briefly contracted to 16.0% and 14.2% respectively, reflecting the deepest period of resource constraint. From 2021 onward, advanced-technique utilization expanded markedly: IMRT first, followed by rapid SBRT and SRS growth, reaching 29.4% in 2021, 34.4% in 2022, 39.6% in 2023, 45.7% in 2024, and 56.8% by 2025. The advanced-technique share continued to rise even during periods of declining total activity (2023), indicating preservation of technical quality within the tertiary center despite systemic stress. Because the relative distribution of primary cancer sites in Lebanon remained stable across 2018–2024 (National Cancer Registry; see Methods), a changing case-mix is unlikely to account for this shift. The persistence of the change after the acute crisis became chronic, rather than a reversion once pressure eased, is more consistent with genuine technique modernization than with a transient, case-mix– or referral-driven artifact, although selective centralization of complex cases at this tertiary center cannot be entirely excluded as a contributing factor.

### 5.3 Workforce gap: Lebanon vs. international benchmarks

Lebanon’s RT workforce falls below ESTRO-HERO benchmarks across key staff categories (**Figure 1**). Radiation therapist availability (3.9 vs. 5–7 per LINAC) and medical physicist availability (0.8 vs. 1.2–1.7 per LINAC) represent the most critical deficits (21, 22). Radiation oncologist staffing (1.4 FTE per LINAC) approaches the lower range of European averages. These structural deficits were present before 2019 and significantly worsened during the economic crisis through brain drain and salary collapse (26).

### 5.4 Temporal acceleration of hypofractionated breast radiotherapy

**Figure 3:**
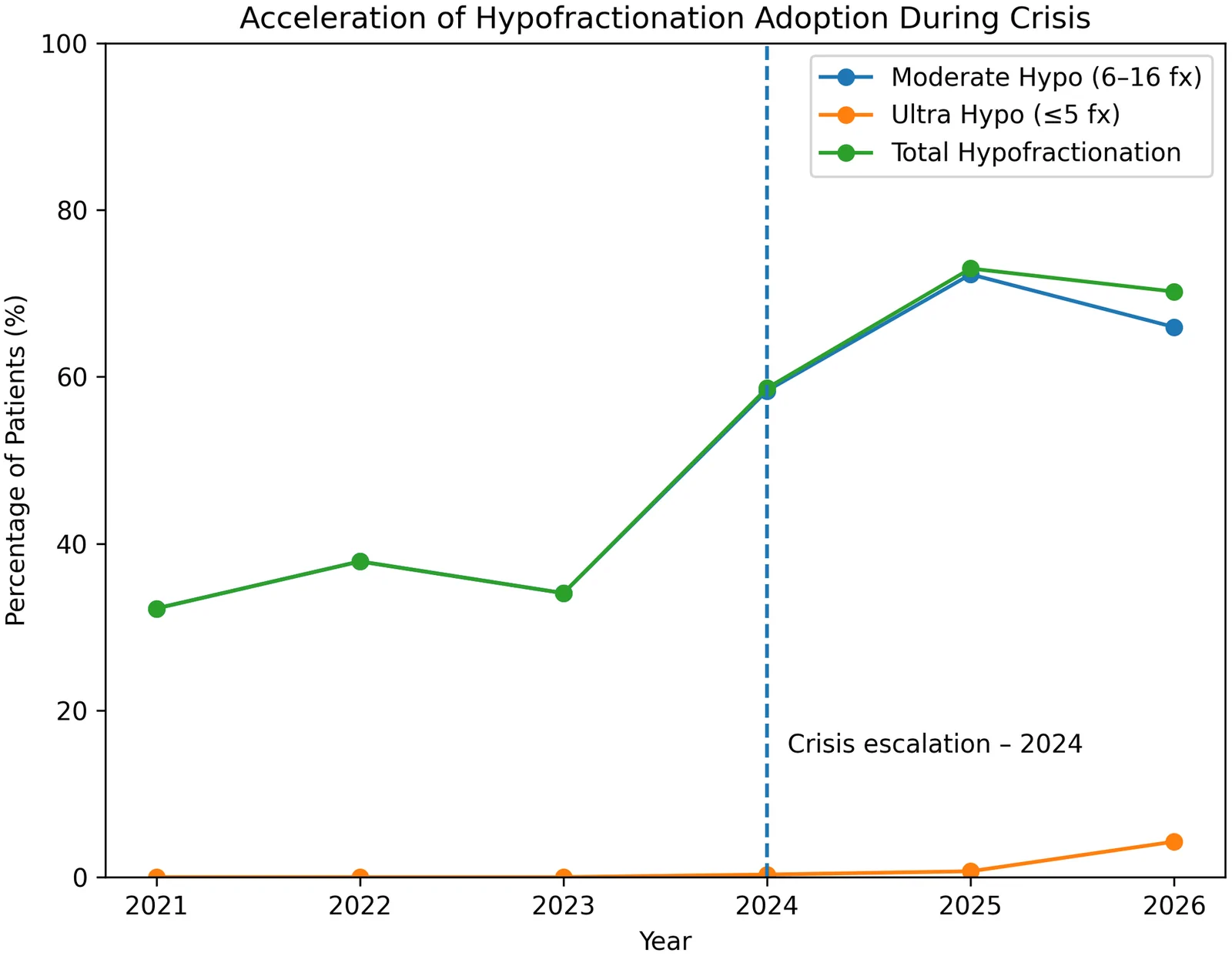
Temporal acceleration of hypofractionated breast radiotherapy at Mount Lebanon Hospital (2021–2026). Annual proportions of patients treated with moderate hypofractionation (6–16 fractions), ultra-hypofractionation (≤5 fractions), and total hypofractionation (≤16 fractions). The dashed vertical line marks 2024, corresponding to the period of armed conflict escalation. Total hypofractionation increased from 34.1% (2023) to 58.6% (2024) and 73.0% (2025).

Between 2021 and 2023, hypofractionated breast radiotherapy (≤16 fractions) demonstrated stable utilization ranging from 32.2% to 37.9% (**Figure 3**). A marked shift began in 2024, with total hypofractionation increasing from 34.1% (2023) to 58.6% (2024) and 73.0% (2025), with sustained levels above 70% in early 2026. Ultra-hypofractionation (≤5 fractions) emerged primarily post-2024, reflecting progressive adoption of shorter evidence-based regimens (33, 34). Once implemented, these strategies persisted beyond the acute crisis, underscoring their role as durable system-level mitigation tools.

## 6 Discussion

### 6.1 Mechanisms of crisis impact on RT services

The Lebanese financial collapse did not affect radiation therapy in isolation; it operated through a cascade of structural and socioeconomic mechanisms. Currency devaluation exceeding 90%, banking collapse, and hyperinflation simultaneously eroded purchasing power, disrupted import-dependent supply chains, and undermined insurance systems (35, 36). Salary erosion accelerated workforce attrition and brain drain, particularly among RTTs and medical physicists (26); while declining educational infrastructure further weakened human resource pipelines. RT, being financially demanding, technology-dependent, and highly workforce-sensitive, proved particularly vulnerable.

**Figure 4:**
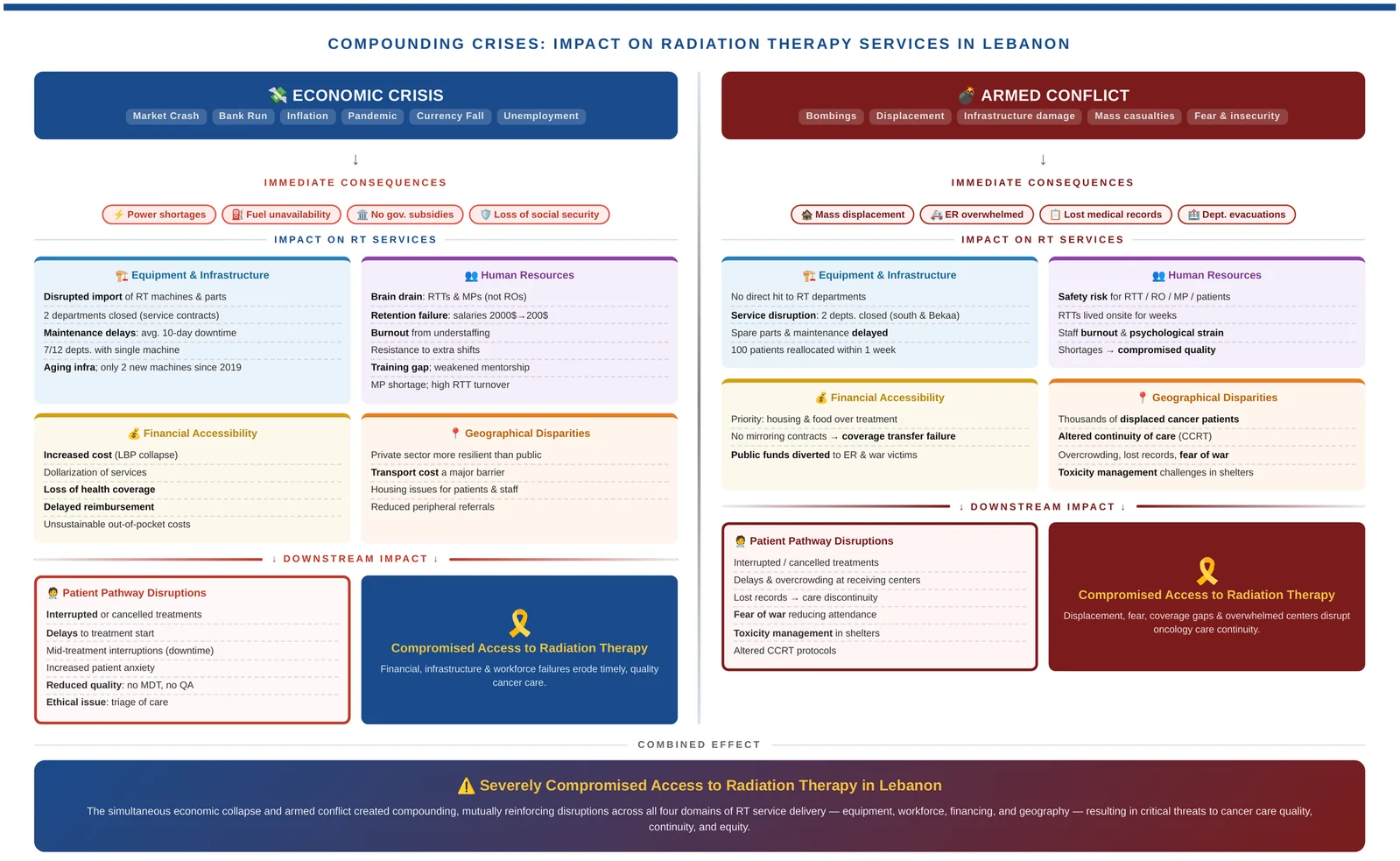
Synergistic impact of economic crisis and armed conflict on radiation therapy service delivery in Lebanon. A dual-column framework illustrating the immediate consequences, impact on RT services across four domains (Equipment & Infrastructure, Human Resources, Financial Accessibility, Geographical Disparities), downstream patient pathway disruptions, and combined effect on RT access for each crisis type. The simultaneous occurrence of both crises produced compounding, mutually reinforcing disruptions across all domains, resulting in critical threats to cancer care quality, continuity, and equity.

While the economic collapse and armed conflict each independently disrupted RT delivery, their combined effect was synergistic rather than additive (**Figure 4**). The economic crisis had already depleted the system’s absorptive buffer before armed conflict imposed acute stressors: displacement, record loss, infrastructure insecurity, and emergency reprioritization (31, 37, 52, 53). These mutually reinforcing breakdowns across equipment, human resources, financing, and patient access converged to push RT access toward a critical vulnerability threshold.

### 6.2 Early numerical stability does not indicate resilience

Early numerical stability or growth in RT activity during crises does not indicate resilience; it may mask progressive erosion of workforce capacity, maintenance buffers, and institutional sustainability, particularly in systems where the majority of departments are private. The compensatory surge during 2019–2022 reflected delayed pricing rescaling (23), drug shortages shifting paradigms toward RT-based strategies, and reduced access to competing systemic therapies. In this study, resilience is conceptualized as a multidimensional property encompassing the capacity to maintain access, treatment quality, workforce function, and equipment continuity, and to recover after shock. Of these dimensions, only sustained treatment volume, an observable proxy for throughput, could be reliably quantified with the available institutional data, whereas treatment quality, interruption rates, and equity of access could not. This distinction is itself central to our argument: because volume captures only one dimension, its apparent stability must not be equated with system resilience. Consistent with this, national cancer incidence rose over the study period while treated volume at MLH grew only modestly and largely through war-related centralization onto a fixed machine base rather than through capacity expansion. This divergence is consistent with a widening gap between need and delivered care that aggregate volume figures alone would conceal; as a single-center observation set against national incidence, however, it cannot establish a national access gap directly. Importantly, the relative stability of treatment volume at MLH should not be interpreted as evidence of national resilience. As a private tertiary center, MLH accepted a broad range of third-party payers, including government-affiliated schemes, and maintained comparatively low out-of-pocket costs, affording it unusual financial agility during currency collapse. This agility, together with the closure of public and government-affiliated centers whether through financial insolvency or, in the south, through armed conflict, positioned MLH to absorb patients redistributed from contracting or closing services. Its maintained volume therefore reflects centralization onto a resilient private node rather than preservation of national capacity; the apparent stability is, in effect, the visible surface of an underlying national contraction. This also refines the comparison with Ukraine: whereas the Ukrainian radiotherapy system is delivered predominantly through public oncology hospitals under national health coverage (with only a small private radiotherapy sector concentrated in advanced modalities) and its disruption and recovery were closely tied to state and international support, the Lebanese experience suggests that, under state insolvency, diversified-payer private capacity may prove comparatively shock-resistant while publicly dependent capacity contracts. The locus of resilience and of vulnerability in fragile radiotherapy systems may thus be shaped substantially by financing and ownership structure, a hypothesis warranting direct multi-center investigation.

Paradoxically, the crisis generated several unintended positive effects: RT became financially more accessible following price rescaling, referral patterns improved as patients migrated toward larger and better-equipped centers, and the scarcity of systemic therapies prompted closer multidisciplinary collaboration between oncologists and radiation oncologists.

### 6.3 Crisis-accelerated modernization: hypofractionation as a resilience tool

Crisis conditions paradoxically catalyzed accelerated adoption of hypofractionation. The inflection in 2024, from 34.1% to 58.6% within a single year, contrasts sharply with the gradual international trend: from 5.4% (2004) to 22.8% (2011) in US national data (38), with COVID-19 not consistently accelerating adoption at the institutional level (39). While the adoption of hypofractionation in conflict settings has been described qualitatively, notably in Ukraine (31, 40, 41), quantitative longitudinal data documenting the magnitude and persistence of crisis-accelerated adoption remain scarce, particularly in settings of compounded, overlapping crises rather than a single armed conflict. The trajectory observed at MLH echoes the Ukrainian experience of volume contraction followed by rebound and accelerated technique modernization during war (40, 41); however, whereas Ukrainian modernization was substantially catalyzed by coordinated international support, including donated and newly procured linear accelerators (41), the adaptations described here occurred without comparable external assistance and amid state insolvency, and were achieved through changes in clinical practice using existing equipment rather than capital investment.

During active conflict, hypofractionation offered clear adaptive advantages: fewer hospital visits minimized unsafe travel; shorter schedules reduced interruption risk from displacement and increased throughput under unpredictable conditions — for example, delivering 15 sessions rather than 25 allowed the department to treat more patients per week, a critical operational buffer when machine availability and staff attendance could not be guaranteed day to day (33, 34). Critically, this transition did not deviate from oncologic standards; rather, it accelerated translation of pre-existing high-level evidence into routine practice (34, 42). Once adopted, practices persisted beyond the acute crisis, confirming hypofractionation as a durable system-level resilience tool rather than a transient adaptation (33, 43).

### 6.4 A proposed layered crisis framework and RT-SRS

**Figure 5:**
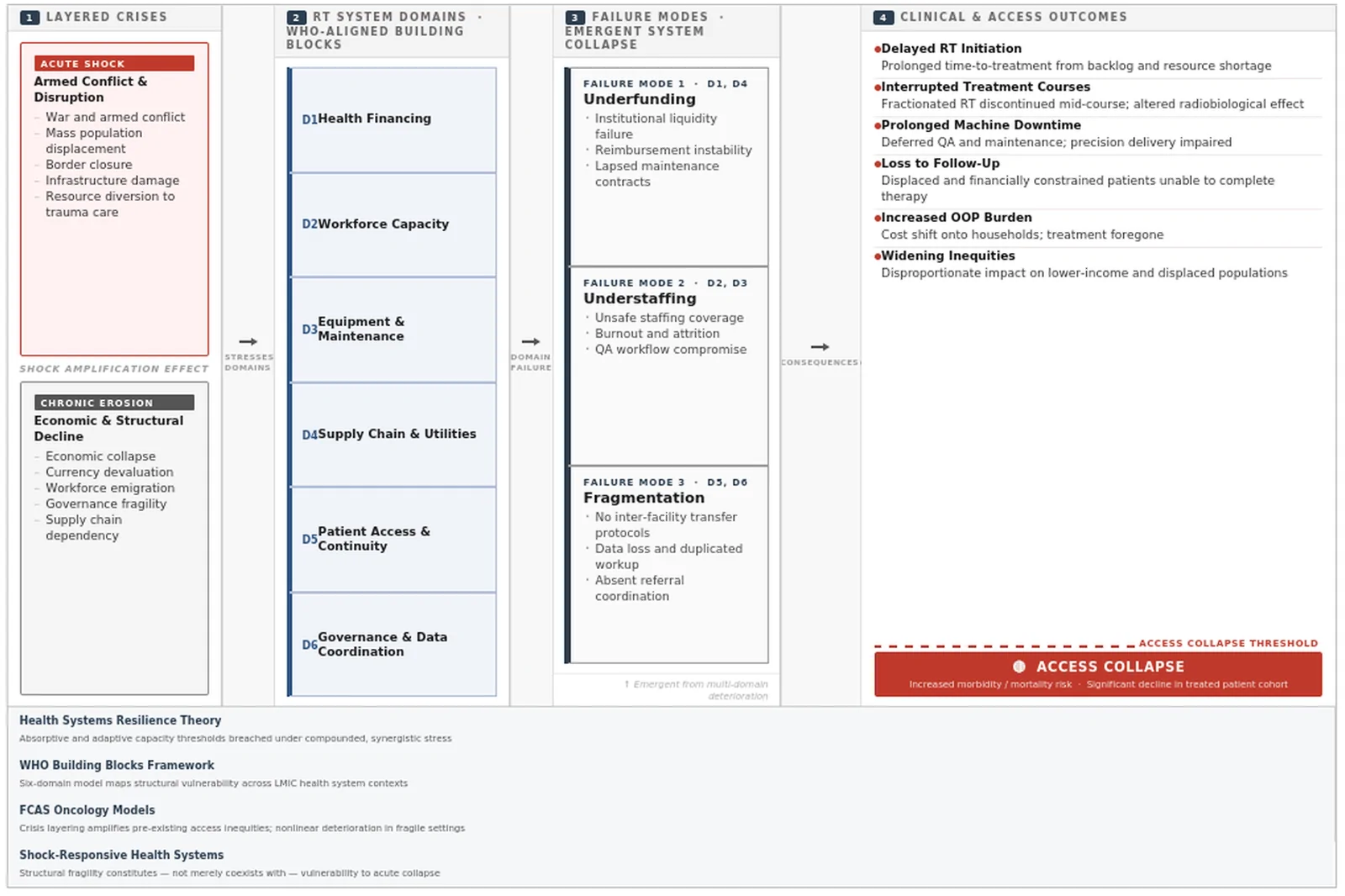
Layered crisis framework for RT system vulnerability and the proposed Radiation Therapy System Resilience Score (RT-SRS). Chronic structural erosion (outer layer) progressively reduces baseline system buffer capacity. Acute shocks propagate stress across six core RT system domains (middle layer), converging into three principal failure modes: underfunding, understaffing, and fragmentation; that generate cascading clinical consequences (inner layer). The access collapse threshold is reached when cumulative stress exceeds absorptive and adaptive capacity. RT-SRS, Radiation Therapy System Resilience Score; LINAC, linear accelerator.

To synthesize these findings into a generalizable structure, we propose the Layered Crisis Framework for RT System Vulnerability (**Figure 5**). This model illustrates how chronic structural erosion progressively reduces baseline system buffer capacity. When an acute shock such as armed conflict occurs, stress propagates across six core RT domains: financing, workforce capacity, equipment and maintenance, supply chain and utilities, patient access and continuity, and governance and data coordination (44, 45).

Multi-domain deterioration converges into three principal failure modes: underfunding, understaffing, and fragmentation, thereby generating cascading clinical consequences. Building on this framework, we propose, as a conceptual, hypothesis-generating contribution rather than a validated instrument, that a structured Radiation Therapy System Resilience Score (RT-SRS) could translate these structural weaknesses into measurable indicators across candidate domains such as workforce stability, equipment redundancy, maintenance capacity, financing continuity, governance readiness, and recovery capacity. The present study identifies the need for such a metric and specifies its candidate domains; it does not develop the score itself. Operationalization will require indicator selection, differential weighting, and threshold definition derived from multi-center data and international evidence through structured expert consensus, together with prospective validation across diverse fragile settings. This requires multi-center data and is therefore beyond the scope of the present single-center study; we designate it as the focus of planned subsequent (Part 2) research. **Figure 5** should therefore be read as a conceptual domain framework rather than as a scoring instrument.

### 6.5 Governance dynamics: cyclical instability rather than linear collapse

**Figure 6:**
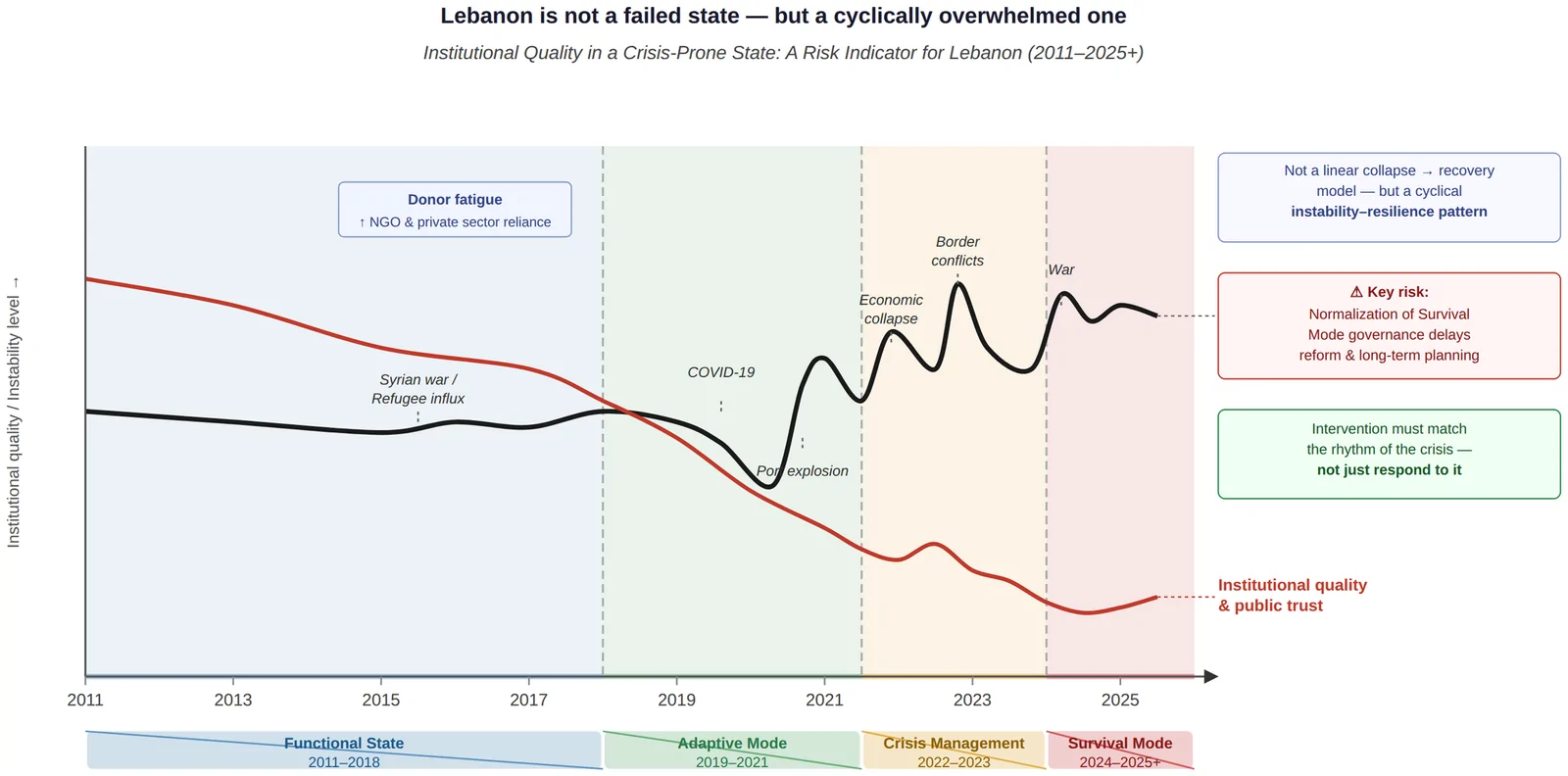
Institutional quality trajectory and governance phase transitions in Lebanon (2011–2025+). The black curve represents an instability tracker illustrating the cyclical stability–instability pattern; the red curve represents institutional quality and public trust (steadily declining). Four governance modes are delineated: Functional State (2011–2018), Adaptive Mode (2019–2021), Crisis Management (2022–2023), and Survival/War Mode (2024–2025+). Key annotated events include Syrian refugee influx, COVID-19, Beirut Port explosion, economic collapse, border conflicts, and armed conflict (2024).

The Lebanese experience reflects a deeper governance dynamic (**Figure 6**). Institutional quality has not followed a linear “collapse–recovery” trajectory, but a cyclical “instability–adaptation” pattern. Periods of functional governance have been followed by adaptive crisis management and prolonged survival-mode administration, with each successive crisis occurring before institutional recovery was consolidated (36, 44). This cyclical pattern normalizes emergency governance and delays structural reform (45). In radiation oncology, this translated into reactive staffing adjustments, temporary mitigation strategies, and reliance on informal networks rather than durable institutional frameworks.

### 6.6 Workforce fragility as the primary bottleneck

Workforce fragility, particularly among RTTs and medical physicists, proved to be the most critical bottleneck, more than equipment availability as a predictor of service disruption (22, 26). This aligns with global evidence that human resource capacity, not machine availability, is the primary constraint on RT access in LMICs (4, 46). Development and implementation of national training and certification programs represents the single most impactful foundational resilience investment available.

## 7 Lessons learned

First, baseline system fragility determines crisis severity. Prolonged economic collapse eroded financing stability, workforce retention, and maintenance capacity before armed conflict escalated. Chronic structural weakness converts shocks into systemic failure.

Second, crisis management alone is insufficient. Sustainable resilience requires certified workforce pipelines, equipment redundancy, interoperable data systems, and protected administrative pathways for spare-part clearance and inter-center patient transfer.

Third, hypofractionation and advanced RT techniques are resilience tools. Embedding these within routine practice and national reimbursement frameworks before crisis onset would substantially enhance RT system resilience (33, 34).

Fourth, individual sacrifice cannot substitute for system design. Systems normalizing prolonged survival-mode governance risk perpetuating long-term dysfunction, quality loss, and workforce exodus.

Fifth, resilience must be measured proactively. A structured RT-SRS capturing workforce stability, equipment redundancy, maintenance capacity, financing continuity, and governance readiness could, once developed and validated, detect vulnerability before access collapse occurs (44, 45).

## 8 Priority solutions

Immediate interventions should include: (1) establishing national RTT and medical physics training and certification programs; (2) implementing formal inter-center mirroring agreements and standardized minimum transfer datasets; (3) introducing fast-track customs clearance for RT spare parts; and (4) leveraging digital tools and telemedicine to partially mitigate workforce shortages.

Although RT is formally recognized within the Lebanese National Cancer Plan, this recognition has yet to be accompanied by the funding, governance structures, and implementation mechanisms required to translate policy into practice. Strengthening RT as a protected essential service during declared emergencies is crucial, requiring systematic data collection on workforce ratios, machine downtime, treatment interruptions, and hypofractionation adoption with formal integration into the National Cancer Registry.

Long-term stabilization requires a structural shift in governance from survival-mode administration to anticipatory system design: reinforcing supply chain resilience, aligning reimbursement with adaptive treatment models, and institutionalizing data-driven planning (32, 47, 49).

## 9 Limitations

This study is observational and primarily descriptive, relying on institutional data from a single tertiary center without patient-level outcome analysis across all national RT facilities. Workforce full-time equivalent calculations include estimated figures and may only partially reflect crisis-related absenteeism^1^. European comparisons are based on published ESTRO-HERO averages and may not account for case-mix complexity. The proposed layered crisis framework and RT-SRS remain conceptual and require prospective validation in diverse fragile settings. Several specific limitations should be made explicit. First, the analysis is single-center; national patient-level RT access cannot be inferred directly, and findings at this referral center are best interpreted as a sentinel-center experience. Second, no clinical outcomes, treatment-interruption rates, or waiting-time data were available. Third, institutional tumor-site distribution over time was not analyzed, although national registry data indicate a stable case-mix across the study period. Fourth, no inferential or time-series statistical modeling was undertaken, so the temporal inflections are descriptive rather than statistically demonstrated. Finally, the Layered Crisis Framework and the RT-SRS are conceptual and have not been operationalized or validated; they are intended as hypothesis-generating tools.

Although centered on a single institution, Mount Lebanon Hospital serves as a national referral center with heterogeneous payer coverage and broad geographic catchment. Its exposure to overlapping economic collapse, infrastructure disruption, workforce attrition, and military escalation reflects multidimensional stressors characteristic of fragile and conflict-affected settings across the world (56).

## 10 Conclusion

Lebanon’s experience demonstrates that radiation therapy systems exposed to compounded crises do not fail linearly, but fluctuate under sustained strain, such that apparent volume stability at a resilient center can mask underlying national contraction. Crisis conditions paradoxically led to rapid adoption of hypofractionation and advanced techniques, resulting in sustainable practice changes that persisted beyond the acute crisis phase. Protecting access to RT in fragile settings will require shifting from reactive crisis-oriented management to proactive health system design anchored by workforce stability, governance reform, adaptive clinical guidelines, and data-driven resilience planning. The above-described Layered Crisis Framework provides a conceptual foundation for this shift, and the proposed RT-SRS offers a structured direction for future operationalization and validation (32, 44, 45).

## Data Availability

The raw data supporting the conclusions of this article will be made available by the authors, without undue reservation.

## Footnotes

1 Crisis-related absenteeism refers to the gap between declared staffing positions and the workforce effectively available for clinical duties, resulting from emigration, salary-driven reduction in working hours, displacement, and burnout. Formal full-time equivalent figures may therefore overestimate functional workforce capacity during crisis periods.

## Supplementary material

Supplementary Figure 1 Geographic distribution of radiation therapy (RT) centers in Lebanon by governorate (2025). Dot size is proportional to the number of linear accelerators (LINACs) per center; color indicates operational status (green: active; yellow: planned; red: closed). The geographic concentration of active centers in the Beirut–Mount Lebanon corridor illustrates the structural centralization of RT services present prior to the 2019 economic crisis, further exacerbated by wartime closure of centers in South Lebanon and the Bekaa during the 2024 armed conflict.
